# Supplementary material for: The Heterostructures of CuO and SnOx for NO2 Detection
Source: Sensors (Basel). 2021 Jun 26;21(13):4387. doi: 10.3390/s21134387 (PMC8272026; doi:10.3390/s21134387)
Supplement: Supplementary file 1 [file sensors-21-04387-s001.zip › sensors-1244298-supplementary.pdf]

Supplementary Materials

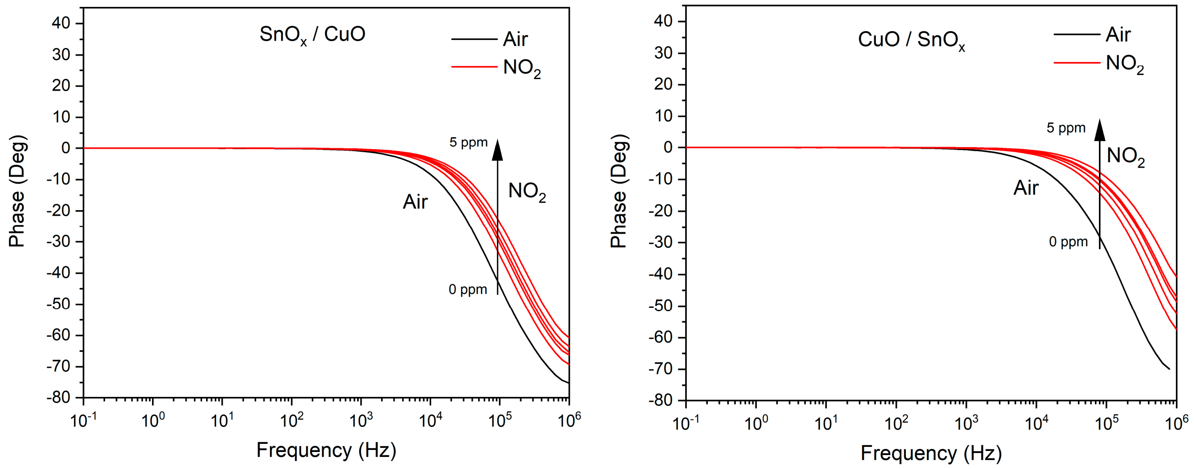

Figure S1. Impedance spectra of the nanomaterials in operating temperature of 275°C in air and upon NO_2 admission (0.5 ppm – 5 ppm): (**a**) Bode representation SnO_x/CuO; (**b**) Bode representation CuO/SnO_x.
